# Effects of Coenzyme Q10 Supplementation on Biomarkers of Oxidative Stress in Adults: A GRADE-Assessed Systematic Review and Updated Meta-Analysis of Randomized Controlled Trials

**DOI:** 10.3390/antiox11071360

**Published:** 2022-07-13

**Authors:** Suming Dai, Zezhong Tian, Dan Zhao, Ying Liang, Meitong Liu, Zhihao Liu, Shanshan Hou, Yan Yang

**Affiliations:** 1 School of Public Health (Shenzhen), Shenzhen Campus of Sun Yat-sen University, Sun Yat-sen University, Shenzhen 518107, China; daism7@mail2.sysu.edu.cn (S.D.); tianzzh@mail2.sysu.edu.cn (Z.T.); zhaod39@mail2.sysu.edu.cn (D.Z.); liangy228@mail2.sysu.edu.cn (Y.L.); liumt7@mail2.sysu.edu.cn (M.L.); liuzhh233@mail2.sysu.edu.cn (Z.L.); houshanshan_33@163.com (S.H.); 2 Guangdong Provincial Key Laboratory of Food, Nutrition, and Health, Sun Yat-sen University, Guangzhou 510080, China; 3 Guangdong Engineering Technology Center of Nutrition Transformation, Sun Yat-sen University, Guangzhou 510080, China; 4 China-DRIs Expert Committee on Other Food Substances, Guangzhou 510080, China

**Keywords:** coenzyme Q10, oxidative stress, total antioxidant capacity, superoxide dismutase, malondialdehyde, meta-analysis

## Abstract

Evidence shows that exogenous CoQ10 supplementation may potentially attenuate oxidative stress status. However, its effective dose and evidence certainty require further evaluation in the general population via more updated randomized controlled trials (RCTs). Databases (PubMed, Embase and Cochrane Library) were searched up to 30 March 2022. Evidence certainty was assessed using the Grading of Recommendations, Assessment, Development and Evaluation (GRADE) approach. Thirty-four RCTs containing 2012 participants were included in this review. Pooled effects of significant increase in total antioxidant capacity (TAC) (standardized mean difference: 1.83, 95%CI: [1.07, 2.59], *p* < 0.001) and significant reduction in malondialdehyde (MDA) concentrations (−0.77, [−1.06, −0.47], *p* < 0.001) were shown after CoQ10 supplementation compared to placebo. However, we could not determine that there was a significant increase in circulating superoxide dismutase (SOD) levels yet (0.47, [0.00, 0.94], *p* = 0.05). Subgroup analyses implied that CoQ10 supplementation was more beneficial to people with coronary artery disease or type 2 diabetes. Additionally, taking 100–150 mg/day CoQ10 supplement had better benefits for the levels of TAC, MDA and SOD (all *p* < 0.01). These results to a statistically significant extent lent support to the efficacy and optimal dose of CoQ10 supplementation on attenuating oxidative stress status in adults.

## 1. Introduction

Oxidative stress, specifically referring to the imbalance between oxidation processes and antioxidant defenses, seems to play a relevant role in the pathogenesis of many age-associated chronic diseases [1,2,3,4,5], and aging or age-associated chronic diseases could also increase the level of oxidative stress [6,7]. The reactive oxygen and nitrogen species (RONS) are highly reactive and toxic molecules continuously produced from the oxidation process [8]. Malondialdehyde (MDA) is the typical product of lipid peroxidation, in which process free radicals attack lipids containing carbon-carbon double bond(s), such as polyunsaturated fatty acids [9]. The body antioxidant defenses contain the enzymatic scavenger of RONS by superoxide dismutase (SOD), which can convert superoxide (O_2_^•−^) into oxygen and hydrogen peroxide [10]. Total antioxidant capacity (TAC), also named nonenzymatic antioxidant capacity, is usually evaluated as the moles of oxidants neutralized by one liter of body fluids [11]. Under the oxidative stress status, the excessive oxidation products suppress the antioxidant defense system of cells, with MDA overproduced and the levels of SOD decreased [11,12,13]. Emerging evidence from long-term prospective studies has suggested that the antioxidant supplementation may be effective in attenuating the outcomes of age-associated chronic diseases [14,15,16].

Coenzyme Q10 (CoQ10) is a lipid-soluble antioxidant mainly biosynthesized by the body itself [17]. In vivo, CoQ10 is present in the inner membrane of mitochondria as an electron carrier where it contributes to oxidative phosphorylation by transporting electrons from complex I and II to complex III [18]. Apart from this, CoQ10 also obtains much attention from its capability of neutralizing free radicals in lipid structures [19]. Although CoQ10 can be endogenously biosynthesized, the production of CoQ10 declines with aging, especially for people with age-associated chronic diseases [20]. In the light of the fact that only minor proportion of CoQ10 is obtained from our diet, the administration of CoQ10 supplements warrants more consideration [21].

Prior systematic reviews of randomized clinical trials (RCTs) have focused on the effects of CoQ10 supplementation on oxidative stress status, but there was less study extended to the general population [22,23,24,25,26]. The number of studies included for the same oxidative stress biomarkers was inconsistent, although the search deadlines were very close [27,28]. Furthermore, less information on evidence quality and evidence certainty was made to ascertain potential clinical translatability and effective dose of CoQ10 supplementation targeting oxidative stress. In this context, a comprehensive systematic review and meta-analysis including more RCTs based on the GRADE (Grading of Recommendations Assessment, Development and Evaluation) method are needed to evaluate the efficacy and optimal dose of CoQ10 supplementation in improving oxidative stress status. 

Therefore, we employed this updated systematic review and meta-analysis with the objective to evaluate the role and effective dose of CoQ10 supplements on oxidative stress biomarkers such as TAC, SOD and MDA in the general population. Furthermore, we assessed the evidence certainty of the antioxidant effect of CoQ10 based on the GRADE approach.

## 2. Materials and Methods

The study followed the guideline of the Preferred Reporting Items for Systematic Reviews and Meta-analyses (PRISMA) protocol for conducting systematic reviews and meta-analyses normatively [29]. This systematic review has been registered on PROSPERO. The registration number is CRD42021252933.

### 2.1. Search Strategy

We searched online databases including PubMed/Medline, Embase and Cochrane library database for the time period until 30 March 2022. To comprehensively find RCTs on the effects of CoQ10 supplementation, we used the following terms in our search strategy: ((Coenzyme Q10) OR (CoQ10) OR (Ubiquinone)) AND ((malondialdehyde) OR (superoxide dismutase) OR (MDA) OR (SOD) OR (total antioxidant capacity) OR (TAC)) (see Appendix A Table A1). The search was restricted to studies published in the English language. 

### 2.2. Selection Criteria

Inclusion was given to studies meeting all of the following criteria: (1) RCTs with a parallel or crossover design; (2) use of a determined amount of CoQ10 intervention; (3) the intervention duration lasting for at least 14 days; (4) available data regarding the pre- and postintervention or changed levels of TAC, MDA and SOD; (5) the control group received placebo or other suitable controls. Studies were excluded if they contained one or more of the following characteristics: (1) acute feeding trials; (2) studies on the pregnant or breastfeeding women; (3) trials with a multifactorial design.

### 2.3. Data Extraction and Quantitative Synthesis

Two investigators (D.Z. and Y.L.) undertook data extraction and quantitative synthesis independently. Confronted with differences of opinions, all the authors partook in a discussion to reach consensus. Extracted data contained the following information: title, the first author, year of publication, study location, study design (parallel or crossover), sample size (intervention and control), study duration, intervention characteristics (form, daily dose), participant characteristics (age, sex and health status), and changes of TAC, MDA and SOD levels. For both the intervention group and control group, the changes of the three biomarkers above were calculated by final mean values minus baseline mean values. Standard deviations (SDs) of the mean difference were obtained by the calculation formula: SD = square root [(SD-baseline value)^2^ + (SD-final value)^2^ − 2R × (SD-baseline value) × (SD-final value)], assuming the R of 0.5. For trials not reporting the SD values or even mean values, we calculated them from available figures or data including standard error of the mean (SEM), median and range by the reliable formula [30]. Of note, data extraction of crossover trials was based on the first intervention period.

### 2.4. Data Analysis

To pool the effect sizes of CoQ10 supplementation, the standardized mean differences (SMDs) and 95%CIs were used because the data extracted could not convert to a uniform unit. The I-square ($I^{2}$) statistic and Cochran’s Q test were performed to assess the heterogeneity between the included studies. $I^{2}$ > 50% and *p*-values < 0.05 were recognized as substantial heterogeneity and significance. Under this circumstance, the random-effects model approach of DerSimonian–Laird was adopted to estimate the overall effects of CoQ10 supplementation on oxidative stress biomarkers. Otherwise, the fixed-effects model with the method of inverse variance was utilized.

Considering that the study duration, intervention dose, health status of participants, control type and study quality may have an association with the net changes of circulating TAC, MDA and SOD levels, we conducted subgroup analyses based on these prespecified variables. Furthermore, the other objective of subgroup analysis was to recognize the potential sources when there was a great heterogeneity between studies. To explore the robustness of the overall effects, we conducted a sensitivity analysis, eliminated the trials one by one and reassessed the overall estimation of the effect. Additionally, the possibility of publication bias was assessed through Egger’s test and inspecting the symmetry of funnel plots. All the statistical analysis was conducted using STATA, version 16.0 (StataCorp, College Station, TX, USA), and R software, version 4.1.2 (http://www.r-project.org/, accessed on 15 March 2022). For all analyses, *p*-values < 0.05 was considered statistically significant. 

### 2.5. Quality Assessment

Two investigators (S.D. and Z.L.) assessed the risk of bias of studies using the Cochrane risk-of-bias tool. It covers seven domains to assess the study bias, which contains random sequence generation, allocation concealment, blinding of participants and personnel, blinding of outcome assessment, incomplete outcome data, selective reporting and other potential bias. Terms including “low”, “unclear” or “high” were given to represent the risk of each domain. A trial owning the result of at least four domains in low risks was considered a relatively good quality. Once a high risk existed, the trial was classified as bad quality. The other trials were thought as fair quality.

### 2.6. Certainty Assessment

The overall certainty of evidence was evaluated using the GRADE (Grading of Recommendations Assessment, Development and Evaluation) method [31]. According to the corresponding evaluation criteria, the effect estimates of oxidative stress biomarkers were graded into four levels, including high, moderate, low, and very low quality.

## 3. Results

### 3.1. Search Flow

**Figure 1:**
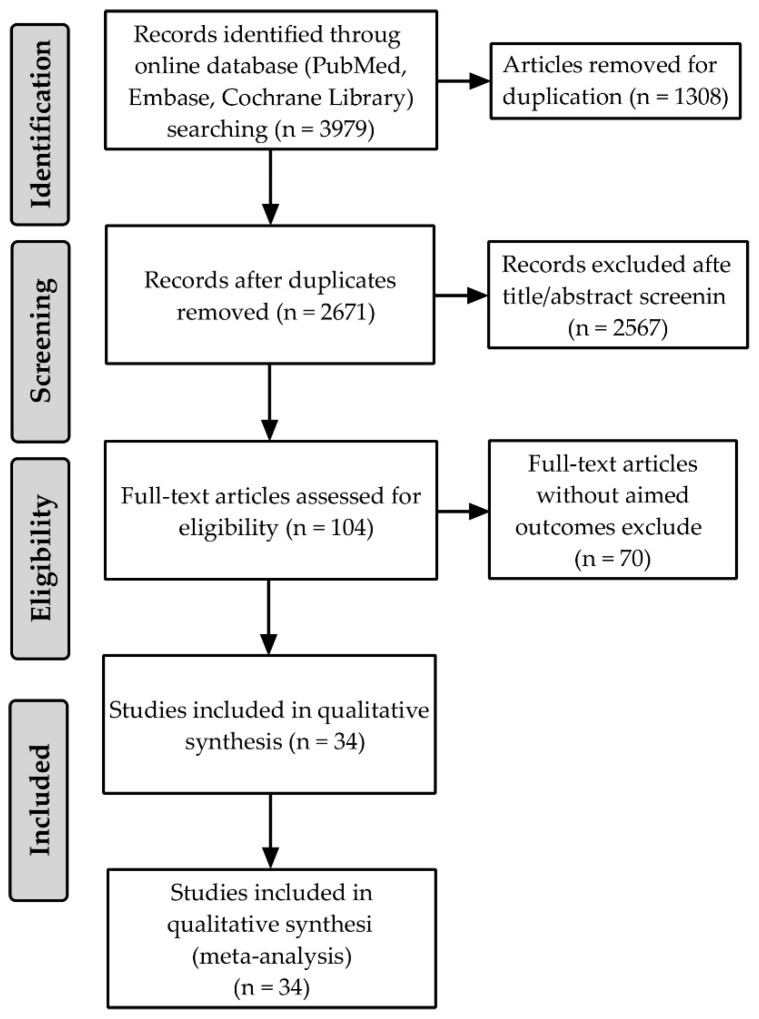
Flow chart of systematic literature search for RCT, published through March 2022, that met the study inclusion and exclusion criteria.

A total of 3979 studies were identified through the initial database search, 1308 of which were removed for duplication. After scanning through title and abstract, 2567 records were excluded. Correspondingly, we assessed the remaining 104 articles for eligibility. Based on the detailed reading of full texts, we finally included 34 RCTs in our analyses. The flow chart of systematic literature search for RCTs that met the study inclusion and exclusion criteria is shown in Figure 1.

### 3.2. Study Characteristics

**Figure A1:**
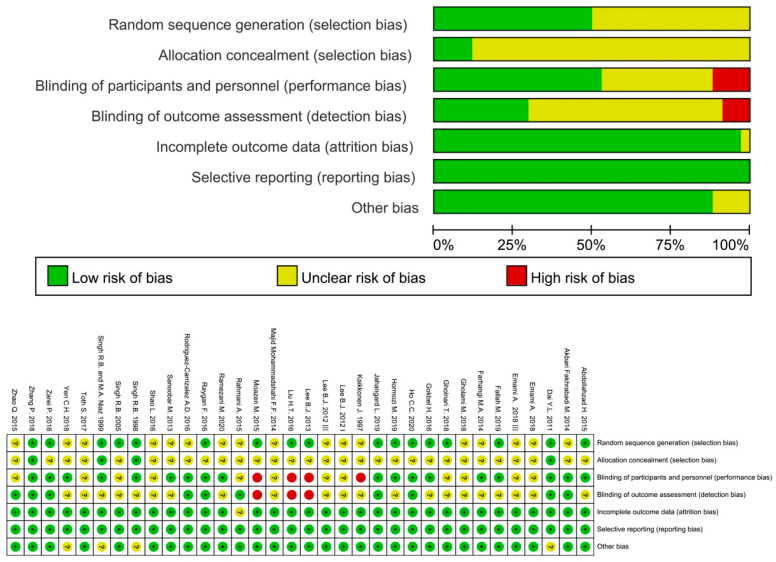
Risk of bias assessment for the included studies. Trials with a low or high risk of bias in key domains were categorized as having a “low risk of bias” or “high risk of bias”, respectively. Otherwise, trials were categorized as having an “unclear risk of bias”.

The characteristics of each included RCT are shown in Table 1. We collected data from 34 studies (including 39 arms) between 1997 and 2020. Up to 32 (94%) studies applied a parallel design. Over half of the studies were performed using participants with cardiovascular or metabolic diseases. Sample sizes varied from 18 to 144. In total, 2012 participants were engaged with these trials, where 1017 subjects were randomly allocated to CoQ10 related intervention group and the remaining 995 were in control group. The range of mean age among these participants was from 17.2 to 79.2 years. Five studies only contained male subjects in healthy status or special occupation [32,33,34,35,36]. Conversely, there were two studies conducted among women only [37,38]. Overall, the 39-arm trials comprised a roughly similar number of male and female subjects, although two trials did not describe the gender distribution of subjects. Daily dose of CoQ10 intervention ranged from 30 mg to 500 mg. The duration of trials lasted from 14 days to 12 months. The summary of risk bias assessment on these included studies is shown in Appendix A Figure A1.

### 3.3. Effect of CoQ10 Supplementation on Circulating TAC

**Figure 2:**
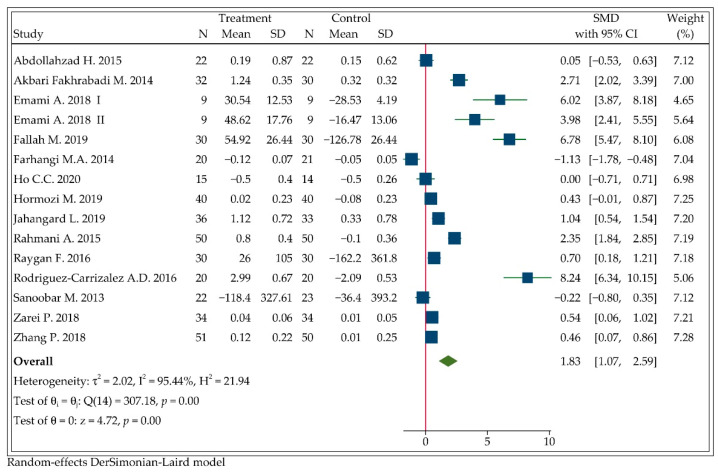
Forest plot of the meta-analysis on the effect of CoQ10 supplementation on net changes of TAC.

**Figure A2:**
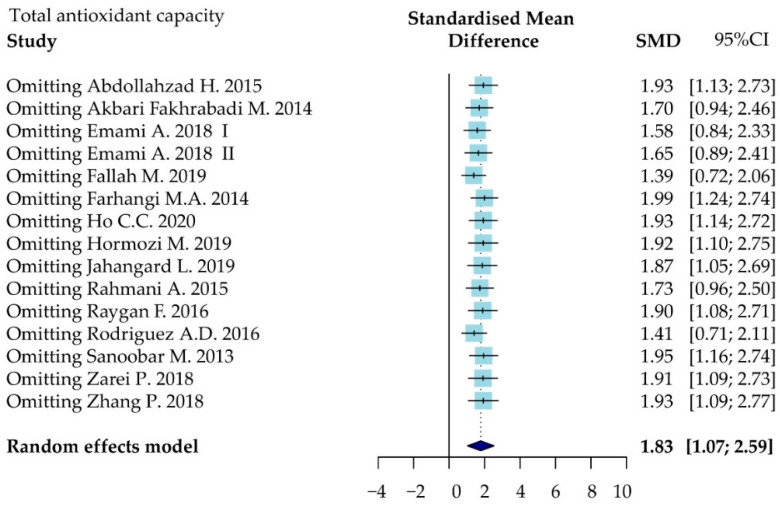
Sensitivity analysis for the effects of CoQ10 on TAC through removal of individual trials one by one.

Fourteen studies with a total of 835 participants measured the circulating TAC after following the CoQ10 supplementation. Emami, A. et al. [33,34] conducted the trial in four parallel groups; thus we included CoQ10 + precooling versus precooling and CoQ10 versus placebo as the results of two independent studies. Hence, a forest plot exhibiting the pooled effect of fifteen arms is presented in Figure 2. TAC levels were significantly increased in participants treated with CoQ10 compared with placebo or others (SMD: 1.83, 95%CI: 1.07 to 2.59, *p* < 0.001). However, there was considerable heterogeneity between the studies ($I^{2}=95.44\%,$ *p* < 0.001). Sensitivity analysis showed that removal of individual studies one by one also did not change the results (see Appendix A Figure A2). 

As mentioned above, we conducted subgroup analysis on prespecified variables to explore the source of heterogeneity and to evaluate the association of them with the overall effect. The corresponding results are shown in Table 2. Among all the participants with different health status, the significant increase in TAC levels still existed in the subsets of disease type including type 2 diabetes (T2D) (SMD: 3.63, 95%CI: 0.82 to 6.45, *p* = 0.01) and others (SMD: 0.73, 95%CI: 0.02 to 1.45, *p =* 0.045). No significant effect was observed in the subset of healthy people (SMD: 3.23, 95%CI: −0.51 to 6.98, *p* = 0.09). In a subgroup analysis on intervention duration, studies administering CoQ10 supplementation for <4 weeks (SMD: 4.86, 95%CI: 2.88 to 6.85, *p* < 0.001), 8–12 weeks (SMD: 0.57, 95%CI: 0.18 to 0.96, *p* < 0.01) and 12–16 weeks (SMD: 1.87, 95%CI: 0.19 to 3.56, *p* = 0.03) showed a significant increase in TAC levels, but the increase was insignificant in the subsets of studies lasting for 4–8 weeks (SMD: 0.61, 95%CI: −2.80 to 4.02, *p =* 0.73) or lasting ≥16 weeks (SMD: 4.30, 95%CI: −3.33 to 11.92, *p =* 0.27). There was also a significant difference (*p* between subgroups *=* 0.02) among the subsets of studies administering 100 mg/d CoQ10 (SMD: 0.26, 95%CI: −0.41 to 11.92, *p =* 0.44), studies administering 100–150 mg/d CoQ10 (SMD: 2.36, 95%CI: 0.72 to 4.00, *p* < 0.01), studies with 150–300 mg/d supplementation dose (SMD: 3.01, 95%CI: 0.82 to 5.21, *p* < 0.01) and studies with ≥300 mg/d supplementation dose (SMD: 3.96, 95%CI: −4.34 to 12.25, *p =* 0.35). 

The heterogeneity had a significant reduction in subgroups of studies administering CoQ10 supplementation for <4 weeks ($I^{2}=55.77\%,$ *p* = 0.13) or 8–12 weeks ($I^{2}=57.93\%,$ *p* = 0.07) but still presented significance across other subgroups. 

### 3.4. Effect of CoQ10 Supplementation on MDA Concentrations

**Figure 3:**
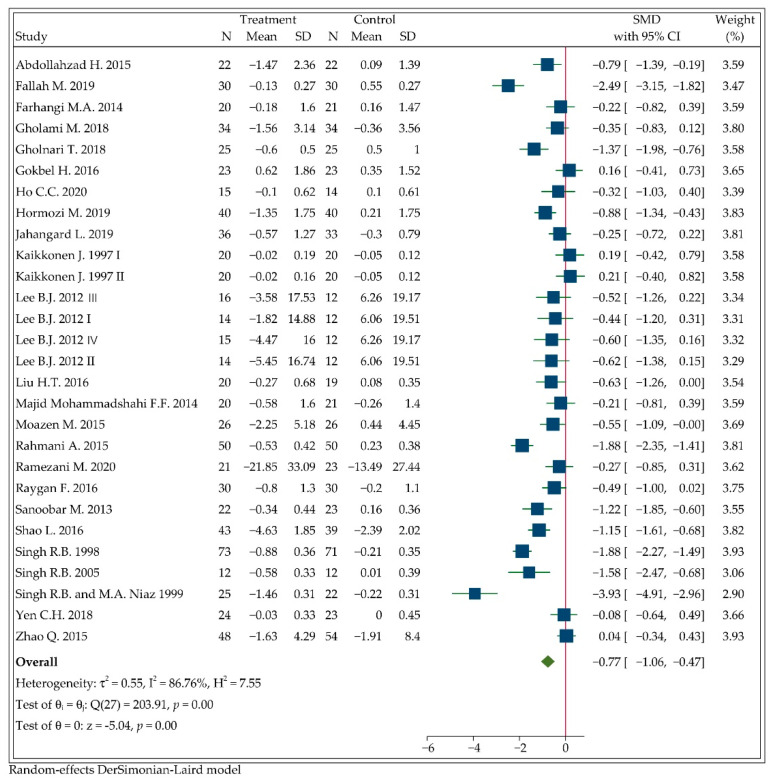
Forest plot of the meta-analysis on the effect of CoQ10 supplementation on net changes of MDA.

**Figure A3:**
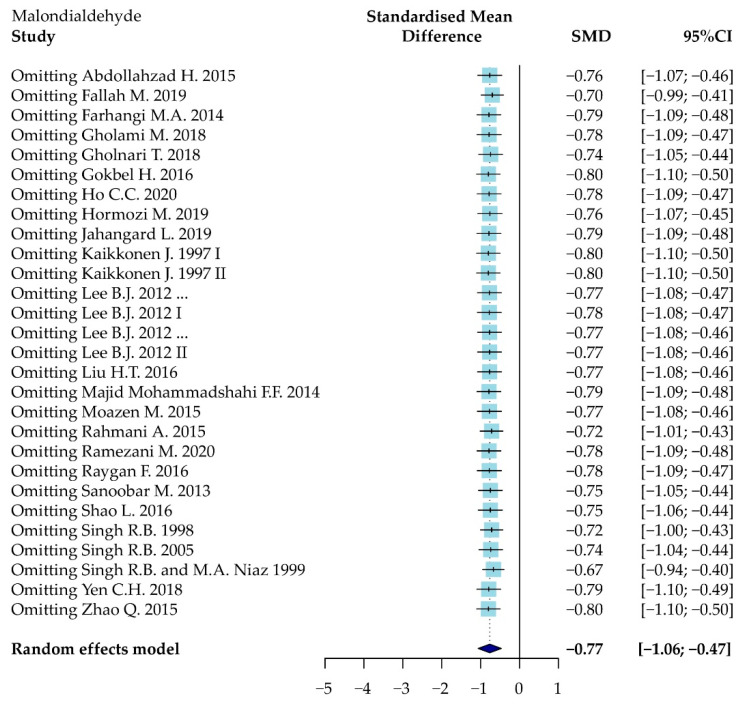
Sensitivity analysis for the effects of CoQ10 on MDA through removal of individual trials one by one.

After combining the results of 28 arms from 25 trials, including a total sample size of 1501 participants, we found a significant effect of CoQ10 intervention on circulating MDA levels using the random-effects model (SMD: −0.77, 95%CI: −1.06 to −0.47, *p* < 0.001; Figure 3). Heterogeneity is significant ($I^{2}=86.76\%,$ *p* < 0.001). The overall effect was robust when we omitted individual study effects one by one for sensitivity analysis (see Appendix A Figure A3).

The results of subgroup analyses in Table 3 showed that the effect of CoQ10 on circulating MDA concentrations could be associated with study duration, intervention dose and participant health status. In subgroups divided by different study duration, the decrease of MDA concentrations remained significant in each subset. In terms of intervention dose, combined results from nine studies administering 100 mg/d CoQ10 supplementation did reveal a significant reduction (SMD: −0.46, 95%CI: −0.71 to −0.22, *p* < 0.001) in MDA concentrations with lower heterogeneity ($I^{2}=42.51\%,$ *p =* 0.08). In the subset of studies with 100–150 mg/d CoQ10 supplementation, the significant reduction still existed (SMD: −1.72, 95%CI: −2.38 to −1.05, *p* < 0.001). However, there was no significant reduction in MDA concentrations in the subset of studies administering <100mg or 150–300 mg CoQ10 supplementation. As for health status, we divided it as detailed as possible. It demonstrated that CoQ10 supplementation significantly reduced MDA concentrations in patients with coronary artery disease (CAD) with no statistical heterogeneity (SMD: −0.55, 95%CI: −0.92 to −0.17, *p =* 0.01; $I^{2}=0.00\%,$ *p =* 0.99), as it did in patients with T2D (SMD: −0.33, 95%CI: −0.64 to −0.03, *p =* 0.03; $I^{2}=0.00\%,$ *p =* 0.49). The significant reduction also existed in patients with acute myocardial infarction (AMI), diabetes nephropathy (DN) or other diseases but was not observed in healthy population or nonalcoholic fatty liver disease (NAFLD) patients, as presented in Table 3.

No matter the type of control, the significant decrease in MDA concentrations was still kept in two subgroups. Additionally, a higher study quality may be associated with a more significant effect of CoQ10 supplementation on MDA levels. Furthermore, subgroup analyses revealed that duration of CoQ10 supplementation, intervention dose, health status of participants and study quality are the possible sources of heterogeneity.

### 3.5. Effect of CoQ10 Supplementation on SOD Levels

**Figure 4:**
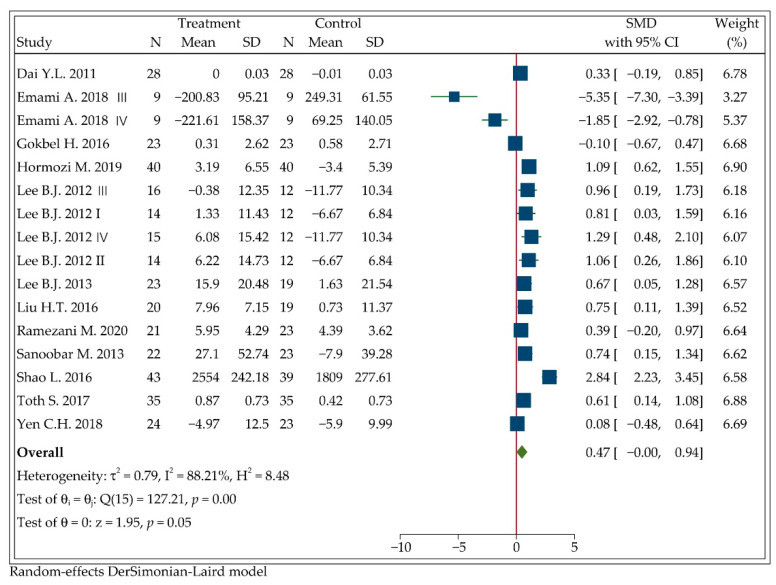
Forest plot of the meta-analysis on the effect of CoQ10 supplementation on net changes of SOD.

**Figure A4:**
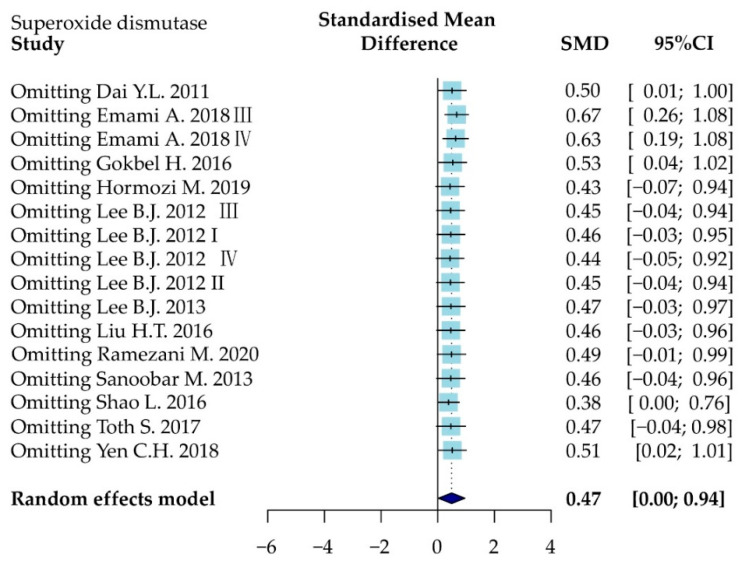
Sensitivity analysis for the effects of CoQ10 on SOD through removal of individual trials one by one.

Based on the pooled results of all 16-arm studies including 694 participants, we could not yet determine that there was a significant increase in SOD levels after following the CoQ10 supplementation (SMD: 0.47, 95%CI: 0.00 to 0.94, *p =* 0.05; Figure 4). Sensitivity analysis demonstrated that after individually eliminating studies by Dai Y.L., Emami A., Gokbel H., Shao L. or Yen C.H. [33,47,52,53,63], the pooled effects of CoQ10 on SOD levels showed a significant elevation, as shown in Appendix A Figure A4. 

In addition, results of subgroup analyses are presented in Table 4. Ten of the included studies evaluated the change of SOD levels after following 12-week CoQ10 supplementation. The combined result of the ten studies showed a significant increase in SOD levels with significantly low heterogeneity (SMD: 0.63, 95%CI: 0.37 to 0.89, *p* < 0.001; $I^{2}=38.80\%,$ *p =* 0.10). Pooling the results of studies in other duration categories, we could not find the significant increase in SOD levels. Following the 100–150 mg/d intervention dose, SOD levels significantly elevated with no statistical heterogeneity between studies (SMD: 1.12, 95%CI: 0.76 to 1.48, *p* < 0.001; $I^{2}=0.00\%,$ *p =* 0.90), while in studies with a higher dose (150–300 mg/d), such significance was eliminated (SMD: −0.18, 95%CI: −0.84 to 0.47, *p =* 0.58). Classifying the studies by different health statuses, we found that CoQ10 supplementation could significantly improve SOD levels in patients with CAD (SMD: 0.92, 95%CI: 0.59 to 1.25, *p* < 0.001). Only Emami A. et al. measured the effect of CoQ10 supplementation on SOD levels of healthy people. After combining the results of this two-arm study, we failed to find a significant change of SOD levels (SMD: −3.50, 95%CI: −6.92 to −0.08, *p =* 0.05). It was also revealed that circulating SOD levels were significantly increased in the subgroup of placebo control (SMD: 0.52, 95%CI: 0.19 to 0.85, *p* < 0.01). Compared to fair-quality studies, the combined effect of good-quality studies was significant with lower heterogeneity (SMD: 0.43, 95%CI: 0.07 to 0.80, *p =* 0.02).

The heterogeneity reduced obviously in three subgroups (12–16 weeks intervention duration, 100–150 mg/d intervention dose and CAD patients). Apart from them, the heterogeneity still remained significant.

### 3.6. Publication Bias

**Figure 5:**
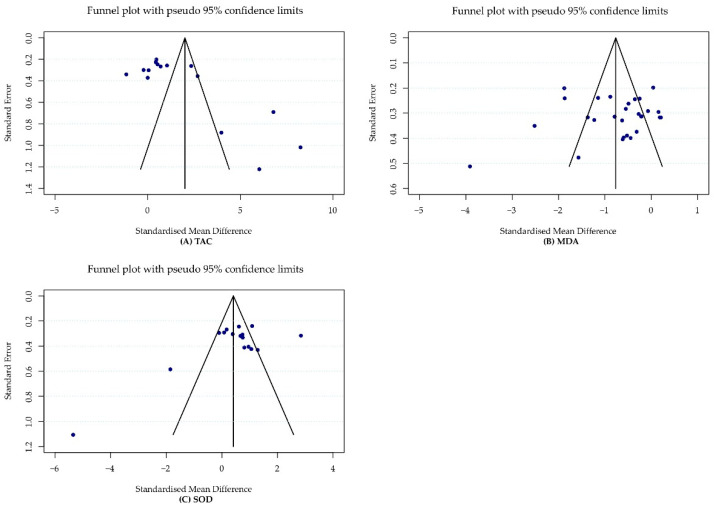
Funnel plots representing publication bias in the included studies relevant to the effect of CoQ10 supplementation on (**A**) TAC, (**B**) MDA and (**C**) SOD.

According to Egger’s test, we found no evidence of publication bias in studies examining the effects of CoQ10 supplementation on MDA (*p =* 0.64) and SOD (*p =* 0.12) levels. However, there was significant publication bias for TAC levels (*p =* 0.01). The results were also visually confirmed by funnel plots (Figure 5).

### 3.7. Grading of the Evidence

The summary of the GRADE assessment of CoQ10 supplementation on the three oxidative biomarkers is shown in Appendix A Table A2. The evidence assessment for TAC, MDA and SOD was all downgraded to very low quality as a result of very serious inconsistency (high heterogeneity) and imprecision (relatively small sample size). Specifically, the evidence estimate of TAC was downgraded to very low quality for potential publication bias.

## 4. Discussion

The current systematic review and meta-analysis quantified the effects of CoQ10 supplementation on oxidative stress biomarkers in 34 RCT comparisons including 2012 participants. The major findings of this review were that CoQ10 supplementation was associated with a significant increase in circulating TAC and a significant reduction in circulating MDA concentrations but only with a border significance in the improvement of SOD levels in general population.

Our results for the effects of CoQ10 supplementation on TAC and MDA levels were in line with those of previous meta-analyses including fewer RCTs, which reported a statistically significant increase in TAC and a significant decrease in MDA levels [27,28,66]. However, the effect of CoQ10 on SOD levels was different from the results of prior meta-analyses that suggested a significant elevation of SOD levels after CoQ10 supplementation. Notably, our review contained 10 additional RCTs for this biomarker that were not included in prior meta-analyses.

Summarizing the included RCTs, we found that the intervention duration in most studies was 12 weeks, followed by 8 weeks. Subgroup analyses on duration suggested that the 12-week CoQ10 supplementation could significantly change the TAC, MDA and SOD levels. Taking CoQ10 for 8 weeks also significantly changed the levels of them except SOD. However, it is worthy to note there were only two RCTs that included evaluating the effect of 8-week CoQ10 supplementation on SOD levels [32,63]. Only three RCTs conducted CoQ10 intervention for more than 12 weeks [55,56,62]; hence we could not conclude that longer intervention time would bring about better antioxidant effects of CoQ10 yet. Subgroup analyses on intervention dose suggested that 100–150 mg/day CoQ10 supplementation was effective to significantly change TAC, MDA and SOD levels at the same time, especially the SOD levels. Consistently, one previous meta-analysis also reported the significant effects of ≤150 mg/day CoQ10 intervention on MDA and SOD levels, although it was specific to people with CAD [23]. Our study more accurately suggested that 100–150 mg/day of CoQ10 had better antioxidant benefits in general population.

In particular, the supplementation with CoQ10 is well-tolerated and safe. All included clinical trials in our present study, with the highest dose 500 mg/d and the longest duration 12 months, showed no side effects causally or plausibly related to CoQ10. Moreover, one 16-month clinical trial using the dose of 1200 mg daily observed no adverse effects [67]. Taking these into account, the recommended 100–150 mg/d of CoQ10 for attenuating the oxidative stress status is considered low risk of side effects.

The potential mechanisms underlying the effects of CoQ10 supplementation on these oxidative stress biomarkers in the general population mainly contain direct and indirect aspects. From the direct aspect, CoQ10 maintains the normal electron transportation in the mitochondrial electron transport chain (METC); thus less superoxide (O_2_^•−^) would be produced in the process [17]. Besides, both in vivo and in vitro studies have suggested that CoQ10 supplementation could ameliorate lipid peroxidation [68,69]. From the indirect aspect, CoQ10 can regenerate α-tocopherol, the reduced active substance of vitamin E, by converting the product from the reaction of vitamin E and lipid peroxidative free radicals [70]. Additionally, CoQ10 could eliminate oxidative stress from the gene aspect by activating nuclear factor erythroid 2-related factor 2 (Nrf-2), a transcription factor regulating cellular responses to oxidative stress through the regulation of a number of ROS-detoxifying enzymes [71,72,73].

Subgroup analyses implied that CoQ10 supplementation was conducive to ameliorating oxidative stress status in patients with T2D or CAD while having no significant effects on oxidative stress biomarkers among healthy counterparts. Concordant with our findings, Jorat et al. reported via the meta-analysis that CoQ10 supplementation significantly increased SOD and decreased MDA levels among patients with CAD [23]. A prior systematic review also found a significant reduction of serum MDA levels after CoQ10 supplementation in a subgroup analysis of diabetic patients [66]. This could be attributed to the decrease of endogenous CoQ10 synthesis in patients with CAD or T2D [74,75], as well as the elevation of circulating CoQ10 concentration after supplementation. For instance, previous studies suggested that CoQ10 levels were significantly lower in T2D patients than those in healthy people and the levels could be restored through exogenous CoQ10 supplementation [76,77]. 

Factors that have been reported regarding the deficiency of CoQ10 include aging and the use of statin-type drugs. In humans, the endogenous production of CoQ10 begins to decline after the age of 20, and the myocardial concentration of CoQ10 is decreased to about half at the age of 80 [20]. Besides, the endogenous synthesis of CoQ10 is suppressed by the extensive use of statin-type drugs in the treatment of several abnormalities linked to CVDs, such as hypercholesterolemia [78,79]. Because the mechanism of statin-type drugs lies in the inhibition of hydroxyl-methylglutaryl coenzyme A (HMG-CoA) reductase, a rate-limiting enzyme acting both in cholesterol synthesis and in the process of CoQ10 biosynthesis [21]. Therefore, low levels of CoQ10 are observed in patients with CVDs or T2D for one or more of the possible factors above. 

This meta-analysis comes with some strengths. The primary strength is that the present study elucidated the association between CoQ10 supplementation and oxidative stress biomarkers in adults through a systematic review and meta-analysis of updated RCTs. Another advantage of this meta-analysis is that we firstly assessed evidence certainty based on the GRADE approach. Nevertheless, our study has some potential limitations. Firstly, due to different measurement methods and different units of these biomarkers, we could not investigate the association between baseline TAC, MDA or SOD levels and the effects of CoQ10 supplementation on them. Also, although our current data indicated that CoQ10 supplementation could attenuate TAC and MDA levels compared to the control group, it is difficult to conclude that CoQ10 could normalize them to the physiological level, which is undefined until now due to the discrepancy of laboratory methods [6]. Secondly, most studies included in this review did not measure participants’ circulating CoQ10 concentrations. Hence, it remains unclear whether circulating CoQ10 status might affect the outcomes explored in this review. Besides, because part of the included studies merely provided the range of age [35,58], we could not make the preplanned subgroup analysis based on the age variable to explore the effects of CoQ10 supplementation varying with the participants’ age. Finally, publication bias may exist in the present study, as in any meta-analysis.

The presence of significant heterogeneity among studies needs to be discussed. An important source of heterogeneity could be due to the discrepancy in laboratory methods used to evaluate the oxidative stress biomarkers. Different techniques on different samples were performed in the included studies. In addition, the formulation of CoQ10 used was various among most included studies, and there was no quantification of CoQ10 intake with diet. Although it was not possible to conclusively ascertain sources of heterogeneity, some implications were given by the subgroup analyses.

## 5. Conclusions

In conclusion, the present systematic review and meta-analysis of 34 RCTs indicates that CoQ10 supplementation may be effective to attenuate oxidative stress status in the general population, especially in people with CAD or T2D. The supplementation of 100–150 mg/day CoQ10 is recommended for ameliorating the oxidative stress status. Further investigations using larger sample size, broad age range of elderly people and longer supplementation period are required to research on the effects of different doses of CoQ10 supplements as well as the circulating CoQ10 levels on people with age-associated chronic diseases.

## Figures and Tables

**Table 1:** Characteristics of included RCTs to investigate the effects of CoQ10 on oxidative stress biomarkers.

First Author, Year	Country	Study Design	Population Features	Sample Size (T/C)	Mean (Age) Years	Sex (Male) %	T	C	Dose mg/day	Duration wk	Biomarkers
Hormozi M. 2019 [32]	Iran	crossover	glazers	80(40/40)	31.83	100.0%	CoQ10	placebo	120	8	TAC, MDA, SOD
Sanoobar M. 2013 [39]	Iran	parallel	multiple sclerosis	45(22/23)	T: 33.1 C: 30.9	8.9%	CoQ10	placebo	500	12	TAC, MDA, SOD
Abdollahzad H. 2015 [40]	Iran	parallel	rheumatoid arthritis	44(22/22)	T: 48.77 C: 50.41	11.4%	CoQ10	placebo	100	8	TAC, MDA
Fallah M. 2019 [41]	Iran	parallel	diabetic hemodialysis	60(30/30)	T: 59.4 C: 64.8	66.7%	CoQ10	placebo	120	12	TAC, MDA
Farhangi M.A. 2014 [42]	Iran	parallel	nonalcoholic fatty liver disease	41(20/21)	T: 42.73 C: 42.18	75.6%	CoQ10	placebo	100	4	TAC, MDA
Ho C.C. 2020 [43]	China	parallel	healthy	29(15/14)	T: 19.9 C: 19.6	69.0%	ubiquinone	placebo	300	12	TAC, MDA
Jahangard L. 2019 [44]	Iran	parallel	bipolar disorder	69(36/33)	T: 37.47 C: 39.52	15.9%	CoQ10	placebo	100	8	TAC, MDA
Rahmani A. 2015 [45]	Iran	parallel	dyspeptic	100(50/50)	T: 57.9 C: 61.0	40.0%	CoQ10	placebo	140	6	TAC, MDA
Raygan F. 2016 [46]	Iran	parallel	obese + T2D + coronary heart disease	60(30/30)	T: 65.9 C: 59.9	unclear	CoQ10	placebo	100	8	TAC, MDA
Gokbel H. 2016 [47]	Turkey	crossover	maintenance hemodialysis	46(23/23)	46.6	15.2%	CoQ10	placebo	200	12	MDA, SOD
Lee B.J. 2012 I [48]	China	parallel	coronary artery disease	26(14/12)	T: 75.1 C: 77.2	92.3%	CoQ10	placebo	60	12	MDA, SOD
Lee B.J. 2012 III [49]	China	parallel	coronary artery disease	28(16/12)	T: 73.0 C: 75.6	92.9%	CoQ10	placebo	60	12	MDA, SOD
Lee B.J. 2012 II [48]	China	parallel	coronary artery disease	26(14/12)	T: 79.2 C: 77.2	96.2%	CoQ10	placebo	150	12	MDA, SOD
Lee B.J. 2012 IV [49]	China	parallel	coronary artery disease	27(15/12)	T: 77.1 C: 75.6	96.3%	CoQ10	placebo	150	12	MDA, SOD
Liu H.T. 2016 [50]	China	parallel	hepatocellular carcinoma	39(20/19)	T: 59.7 C: 61.5	69.2%	CoQ10	placebo	300	12	MDA, SOD
Ramezani M. 2020 [51]	Iran	parallel	acute ischemic stroke	44(21/23)	T: 64.10 C: 62.04	50.0%	CoQ10	placebo	300	4	MDA, SOD
Shao L. 2016 [52]	China	parallel	acute viral myocarditis	82(43/39)	T: 23 C: 25	51.2%	ubiquinol + trimetazidine	other	30	2	MDA, SOD
Yen C.H. 2018 [53]	China	parallel	T2D	47(24/23)	T: 61.5 C: 59.6	66.0%	liquid ubiquinol	placebo	100	12	MDA, SOD
Akbari Fakhrabadi M. 2014 [54]	Iran	parallel	T2D	62(32/30)	T: 56.7 C: 54.8	25.8%	CoQ10	placebo	200	12	TAC
Emami A. 2018 I [34]	Iran	parallel	healthy	18(9/9)	T: 17.40 C: 17.20	100.0%	CoQ10 + precooling	precooling	300	2	TAC
Emami A. 2018 II [34]	Iran	parallel	healthy	18(9/9)	T: 17.60 C: 17.71	100.0%	CoQ10	placebo	300	2	TAC
Rodriguez-Carrizalez A.D. 2016 [55]	Mexico	parallel	T2D	40(20/20)	T: 28.2 C: 29.3	50.0%	CoQ10	placebo	400	24	TAC
Zarei P. 2018 [37]	Iran	parallel	T2D	68(34/34)	T: 53.1 C: 53.35	0.0%	CoQ10	placebo	100	12	TAC
Zhang P. 2018 [56]	China	parallel	dyslipidemia	101(51/50)	T: 51.78 C: 50.02	31.7%	CoQ10	placebo	120	24	TAC
Gholami M. 2018 [38]	Iran	parallel	T2D	68(34/34)	T: 53.1 C: 53.35	0.0%	CoQ10	placebo	100	12	MDA
Gholnari T. 2018 [57]	Iran	parallel	diabetic nephropathy	50(25/25)	T: 61.1 C: 61.6	32.0%	CoQ10	placebo	100	12	MDA
Kaikkonen J. 1997 I and II [36]	Finland	parallel	smoking	60(20/20/20)	46	100.0%	CoQ10	placebo	90	8	MDA
Majid Mohammadshahi F.F. 2014 [58]	Iran	parallel	nonalcoholic fatty liver disease	41(20/21)	19–54 (range)	unclear	CoQ10	placebo	100	12	MDA
Moazen M. 2015 [59]	Iran	parallel	T2D	52(26/26)	T: 50.67 C: 52.79	53.8%	CoQ10	placebo	100	8	MDA
Singh R.B. 1998 [60]	India	parallel	acute myocardial infarction	144(73/71)	T: 48.0 C: 47.6	79.9%	CoQ10	B vitamin	120	4	MDA
Singh R.B. 2005 [35]	India	parallel	healthy	24(12/12)	18–55 (range)	100.0%	CoQ10	placebo	200	20 days	MDA
Singh R.B. and M.A. Niaz 1999 [61]	India	parallel	acute myocardial infarction, unstable angina, angina pectoris	47(25/22)	T: 48.4 C: 47.6	78.7%	CoQ10	placebo	120	4	MDA
Zhao Q. 2015 [62]	China	parallel	heart failure of nonischemic origin	102(48/54)	T: 63 C: 62	70.6%	CoQ10	placebo	30	48	MDA
Dai Y.L. 2011 [63]	China	parallel	ischemic left ventricular systolic dysfunction	56(28/28)	T: 67.7 C: 70.1	92.9%	CoQ10	placebo	300	8	SOD
Emami A. 2018 III [33]	Iran	parallel	healthy	18(9/9)	T: 17.40 C: 17.20	100.0%	CoQ10 + precooling	precooling	300	2	SOD
Emami A. 2018 IV [33]	Iran	parallel	healthy	18(9/9)	T: 17.60 C: 17.71	100.0%	CoQ10	placebo	300	2	SOD
Lee B.J. 2013 [64]	China	parallel	coronary artery disease	42(23/19)	T: 71.7 C: 66.5	73.8%	CoQ10	placebo	300	12	SOD
Toth S. 2017 [65]	Slovakia	parallel	dyslipidemia	70(35/35)	T: 58.4 C: 61.96	50.0%	CoQ10 + omega-3 PUFA	omega-3 PUFA	200	12	SOD

Abbreviations: RCTs, randomized controlled trials; T, treatment group; C, control group; T2D, Type 2 Diabetes.

**Table 2:** Subgroup analyses of CoQ10 supplementation on TAC.

Subgroup	No.	SMD (95%CI)	*p*-Value	$I^{2}$	*p* for Heterogeneity	*p* between Subgroups
**Overall**	15	1.83 (1.07, 2.59)	<0.001	95.44%	<0.001	
**Duration**						
<4 weeks	2	4.86 (2.88, 6.85)	<0.001	55.77%	0.13	0.001
≥4 weeks and <8 weeks	2	0.61 (−2.80, 4.02)	0.73	98.54%	<0.001	
≥8 weeks and <12 weeks	4	0.57 (0.18, 0.96)	<0.01	57.93%	0.07	
≥12 weeks and <16 weeks	5	1.87 (0.19, 3.56)	0.03	96.85%	<0.001	
≥16 weeks	2	4.30 (−3.33, 11.92)	0.27	98.37%	<0.001	
**Intervention dose**						
100 mg/d	5	0.26 (−0.41, 11.92)	0.44	87.01%	<0.001	0.02
>100 mg/d and ≤150 mg/d	4	2.36 (0.72, 4.00)	<0.01	97.37%	<0.001	
>150 mg/d and ≤300 mg/d	4	3.01 (0.82, 5.21)	<0.01	94.41%	<0.001	
>300 mg/d	2	3.96 (−4.34, 12.25)	0.35	98.56%	<0.001	
**Health status**						
DN	1	-				<0.001
Dyslipidemia	1	-				
Healthy	3	3.23 (−0.51, 6.98)	0.09	95.26%	<0.001	
NAFLD	1	-				
T2D	3	3.63 (0.82, 6.45)	0.01	97.35%	<0.001	
Other	6	0.73 (0.02, 1.45)	0.045	91.37%	<0.001	
**Type of control**						
Placebo	14	1.61 (0.86, 2.37)	<0.001	95.44%	<0.001	<0.001
Other	1	-				
**Study quality**						
Fair	3	3.91 (1.86, 5.95)	<0.001	85.20%	0.001	0.02
Good	12	1.37 (0.60, 2.14)	0.001	95.20%	<0.001	

**Table 3:** Subgroup analyses of CoQ10 supplementation on MDA.

Subgroup	No.	SMD (95%CI)	*p*-Value	$I^{2}$	*p* for Heterogeneity	*p* between Subgroups
**Overall**	28	−0.77 (−1.06, −0.47)	<0.001	86.76%	<0.001	
**Duration**						
<4 weeks	2	−1.24 (−1.65, −0.83)	<0.001	0.00%	0.40	<0.001
≥4 weeks and <8 weeks	5	−1.59 (−2.58, −0.60)	<0.01	93.80%	<0.001	
≥8 weeks and <12 weeks	7	−0.39 (−0.70, −0.08)	0.02	57.43%	0.03	
≥12 weeks and <16 weeks	13	−0.66 (−1.04, −0.29)	0.001	77.79%	<0.001	
≥16 weeks	1	-				
**Intervention dose**						
<100 mg	6	−0.28 (−0.76, 0.20)	0.25	76.65%	0.001	0.001
100 mg/d	9	−0.46 (−0.71, −0.22)	<0.001	42.51%	0.08	
>100 mg/d and ≤150 mg/d	7	−1.72 (−2.38, −1.05)	<0.001	89.04%	<0.001	
>150 mg/d and ≤300 mg/d	5	−0.46 (−0.96, 0.03)	0.07	63.93%	0.03	
>300 mg/d	1	-				
**Health status**						
AMI	2	−2.85 (−4.87, −0.84)	0.01	93.21%	<0.001	<0.01
CAD	4	−0.55 (−0.92, −0.17)	0.01	0.00%	0.99	
DN	2	−1.92 (−3.01, −0.83)	0.001	82.93%	0.02	
HF	1	-				
Healthy	4	−0.32 (−1.03, 0.39)	0.38	75.99%	0.01	
NAFLD	2	−0.21 (−0.64, 0.21)	0.33	0.00%	0.99	
T2D	3	−0.33 (−0.64, −0.03)	0.03	0.00%	0.49	
Other	10	−0.75 (−1.13, −0.37)	<0.001	80.24%	<0.001	
**Type of control**						
Placebo	26	−0.70 (−0.10, −0.40)	<0.001	84.68%	<0.001	0.04
Other	2	−1.52 (−2.24, −0.81)	<0.001	81.97%	0.02	
**Study quality**						
Bad	2	−0.58 (−1.00, −0.17)	<0.01	0.00%	0.85	0.58
Fair	11	−0.63 (−1.06, −0.20)	<0.01	81.42%	<0.001	
Good	15	−0.90 (−1.36, −0.43)	<0.001	90.61%	<0.001	

**Table 4:** Subgroup analyses of CoQ10 supplementation on SOD.

Subgroup	No.	SMD (95%CI)	*p*-Value	$I^{2}$	*p* for Heterogeneity	*p* between Subgroups
**Overall**	16	0.47 (−0.00, 0.94)	0.05	88.21%	<0.001	
**Duration**						
<4 weeks	3	−1.38 (−5.85, 3.10)	0.55	98.04%	<0.001	0.71
≥4 weeks and <8 weeks	1	-				
≥8 weeks and <12 weeks	2	0.72 (−0.03, 1.46)	0.06	78.00%	0.03	
≥12 weeks and <16 weeks	10	0.63 (0.37, 0.89)	<0.001	38.80%	0.10	
≥16 weeks	0	-				
**Intervention dose**						
<100 mg	3	1.55 (0.18, 2.93)	0.03	90.91%	<0.001	0.001
100 mg/d	1	-				
>100 mg/d and ≤150 mg/d	3	1.12 (0.76, 1.48)	<0.001	0.00%	0.90	
>150 mg/d and ≤300 mg/d	8	−0.18 (−0.84, 0.47)	0.58	87.15%	<0.001	
>300 mg/d	1	-				
**Health status**						
CAD	5	0.92 (0.59, 1.25)	<0.001	0.00%	0.80	0.02
Dyslipidemia	1	-				
HF	1	-				
Healthy	2	−3.50 (−6.92, −0.08)	0.045	89.41%	<0.01	
T2D	1	-				
Other	6	0.95 (0.18, 1.72)	0.02	90.85%	<0.001	
**Type of control**						
Placebo	13	0.52 (0.19, 0.85)	<0.01	70.31%	<0.001	0.51
Other	3	−0.39 (−3.06, 2.28)	0.78	97.39%	<0.001	
**Study quality**						
Bad	2	0.71 (0.27, 1.15)	<0.01	0.00%	0.86	0.57
Fair	8	0.26 (−0.81, 1.32)	0.63	93.39%	<0.001	
Good	6	0.43 (0.07, 0.80)	0.02	63.31%	0.02	

## Appendix A

**Table A1:** Search strategy. For all databases, searches were performed for the time period until 30 March 2022.

PubMed	#1 ((Coenzyme Q10) OR (CoQ10) OR (Ubiquinone)) #2 ((malondialdehyde) OR (superoxide dismutase) OR (MDA) OR (SOD) OR (total antioxidant capacity) OR (TAC)) #1 AND #2
Embase	#1 ‘coenzyme q10’/exp OR ‘coenzyme q10’ OR coq10 OR ‘ubiquinone’/exp OR ubiquinone #2 ‘malondialdehyde’/exp OR malondialdehyde OR ‘superoxide dismutase’/exp OR ‘superoxide dismutase’ OR mda OR sod OR ‘total antioxidant capacity’/exp OR ‘total antioxidant capacity’ OR tac #1 AND #2
Cochrane Library	#1 ((Coenzyme Q10) OR (CoQ10) OR (Ubiquinone)) #2 ((malondialdehyde) OR (superoxide dismutase) OR (MDA) OR (SOD) OR (total antioxidant capacity) OR (TAC)) #1 AND #2

**Table A2:** GRADE evidence profile of CoQ10 supplementation on oxidative stress biomarkers.

Quality Assessment							No of Patients		Effect	Quality	Importance
No of Studies	Design	Risk of Bias	Inconsistency	Indirectness	Imprecision	Other Considerations	CoQ10	Control	Absolute (95%CI)		
**Total Antioxidant Capacity (follow-up 2–24 weeks; Better Indicated by higher values)**											
15	randomized trials	no serious	very serious ^1^	no serious	very serious ^2^	reporting bias ^3^	420	415	SMD 1.83 higher (1.07 to 2.59 higher)	⊕OOO VERY LOW	CRITICAL
**Malondialdehyde (follow-up 2–48 weeks; Better indicated by lower values)**											
28	randomized trials	no serious	very serious ^4^	no serious	serious ^2^	none	758	743	SMD 0.77 lower (1.06 to 0.47 lower)	⊕OOO VERY LOW	CRITICAL
**Superoxide Dismutase (follow-up 4–12 weeks; Better indicated by higher values)**											
16	randomized trials	no serious	very serious ^5^	no serious	very serious ^2^	none	356	338	SMD 0.47 higher (0.00 to 0.94 higher)	⊕OOO VERY LOW	CRITICAL

^1^ The test for heterogeneity between studies is significant (*p* < 0.001), and the I2 equals 95.44%. ^2^ The sample size is relatively small. ^3^ The Egger’s test for TAC to identify publication bias is significantan (*p =* 0.01). ^4^ The test for heterogeneity between studies is significant (*p* < 0.001), and the I2 equals 86.76%. ^5^ The test for heterogeneity between studies is significant (*p* < 0.001), and the I2 equals 88.21%.
